# Strong cooling induced by stand-replacing fires through albedo in Siberian larch forests

**DOI:** 10.1038/s41598-018-23253-1

**Published:** 2018-03-19

**Authors:** Dong Chen, Tatiana V. Loboda, Tao He, Yi Zhang, Shunlin Liang

**Affiliations:** 1 Department of Geographical Sciences, University of Maryland, College Park, Maryland USA; 2 School of Remote Sensing and Information Engineering, Wuhan University, Wuhan, Hubei China

## Abstract

The Siberian larch forests, taking up about a fifth of the global boreal biome, are different from the North American boreal forests in that they generally do not undergo a secondary succession. While wildfires in the boreal forests in North America have been shown to exert a cooling effect on the climate system through a sharp increase in surface albedo associated with canopy removal and species composition change during succession, the magnitude of the surface forcing resulting from fire-induced albedo change and its longevity in Siberia have not been previously quantified. Here we show that in contrast to previous expectations, stand-replacing fires exert a strong cooling effect similar in magnitude to that in North America. This cooling effect is attributable to the increase in surface albedo during snow-on periods. However, the observed earlier snowmelt in the region, and subsequently a longer snow-free season, has resulted in a warming effect which has the potential to offset the fire-induced cooling. The net albedo-induced forcing of the Siberian larch forests in the future would hinge on the interaction between the fire-induced cooling effect and the climate-induced warming effect, both of which will be impacted by the expected further warming in the region.

## Introduction

The boreal forest is one of the largest biomes in the world^1,2^ and a key element in the global climate system that has the capacity to impact regional and global climate^3^. Its influence on climate is exerted mainly through two sets of mechanisms: the biogeochemical effects, primarily through the carbon cycle, and the biogeophysical effects including albedo, evapotranspiration, and surface roughness. The boreal forest has a strong impact on global temperatures which is largely attributable to the increased absorption of solar radiation caused by the considerably lower albedo of the boreal forest during the snow season, relative to other land cover types such as tundra and bare ground^3,4^. The albedo-driven warming effect likely overshadows the cooling resulting from the carbon sequestration in the boreal zone and is the dominant driver controlling the net climatic impact of the boreal forest^3–6^.

Because albedo of the boreal biome exerts a strong control over climate, any forces that significantly change forest albedo, including wildfire, can potentially carry strong climatic implications. Wildfire is a dominant natural disturbance agent in the boreal forest that controls species composition, stand age, and successional patterns^1,7–10^. Wildfire impacts have been studied extensively in the boreal forests of North America, where wildfires have been found to impose a general cooling effect after fire^11–15^. This is attributable to two factors. First, wildfires in the North American boreal forests are generally stand-replacing. Thus the loss of the forest canopy results in considerably higher albedo during the snow season^11,13^. Second, burned forests recover slowly over a prolonged successional period when forest composition is dominated by deciduous broadleaf species, which typically have higher albedo than needleleaf evergreen species^12,13,16–18^. Randerson *et al*.^11^ constructed a radiative forcing trajectory induced by post-fire albedo changes in the burned black spruce (*Picea mariana*) forest stands in interior Alaska. Their results indicated that albedo-induced radiative forcing in the burned forests became negative one year after fire and remained so for 50 years. The magnitude of the cooling effect increased rapidly in the first 10 years after fire when it peaked at −8 ± 3 Wm^−2^ and then slowly decreased to reach the pre-fire levels 50 years after the fire occurrence. The cumulative cooling effect of the fire-induced albedo changes outweighed the combined warming effect of the CO_2_ and CH_4_ emissions, resulting in a net cooling effect estimated to last for more than 150 years. Rogers *et al*.^14^ also established an albedo-induced surface forcing (SF) trajectory for the burned forests in North America. Based on MODIS data, their results, although limited to an 11-year time span, were well-aligned with those of Randerson *et al*.^11^.

With the total area of about a fifth of the global boreal forest^19^, the forests of Eastern Siberia are an important component of the boreal biome. Underlain by continuous permafrost and dominated by larch (deciduous needleleaf species), these forests are strikingly different from their North American and European counterparts^19,20^. Larch species dominate forest composition at all recovery stages and generally do not undergo secondary broadleaf successional stages. This implies that the knowledge gained about the climatic impact of the fire-induced albedo changes in the North American boreal forests cannot be readily applied to the larch forests of Siberia. A recent study conducted by Rogers *et al*.^14^ indicates that the post-fire stands in boreal Northern Eurasia also produce an albedo-induced cooling effect although of a much smaller magnitude compared to those in the North American boreal forests. However, considering the widespread occurrence of surface fires in Siberia - a major difference in fire regime between the two boreal zones^21^ - a more focused examination of the impacts of wildfires in Siberia is needed. In this study we aim to isolate the impacts of stand-replacing fires within the Siberian larch forests on SF using a recently developed stand age map of the Siberian larch forests^22^ along with the full record of the collection 6 MODIS albedo product^23^. We focus on understanding the temporal trajectory of SF resulting from stand-replacing fires as well as quantifying the impact of those fires on the SF produced by the Siberian larch forests as a whole between 2001 and 2012.

## Data and Methods

### Study area

The study area of the current project is the Siberian larch forests, which is the forests in Eastern Siberia that are dominated by larch. It is located within 50°–66° N and 94°–140° E and the total area is 1.93 × 10^8^ ha. The delineation of the study area was based on the deciduous needleleaf forest class in the 500-m MODIS Land Cover Type Product (MCD12Q1; Friedl *et al*.^24,25^) (see details in Chen *et al*.^22^). Within this region, wildfires are the most dominant disturbance agent, accounting for 87% of forest loss between 2001 and 2012^22^.

### Surface forcing

Daily shortwave black-sky (BSA) and white-sky (WSA) albedo values between 2001 and 2015 within the study area were extracted from the MODIS albedo product (MCD43A3, Collection 6; Schaaf *et al*.^23^) and used to calculate annual mean BSA and WSA. The calculation of the annual mean albedo for each pixel was hierarchical, where the monthly mean values were calculated first, based on which the annual mean values were then calculated. The extremely low solar angles result in very few valid albedo observations between November and February. Consequently, the mean albedo for those months would further hinder the calculation of the annual mean values. To compute the annual mean albedo across the study area, in cases when January and February monthly mean albedo was not available, the mean albedo values of those two months were set to the first valid albedo value in March of the corresponding year (e.g. January and February of 2015 received the mean monthly value of March 2015). Similarly, the mean albedo values for November and December, were set to the last valid albedo observation in October (e.g. November and December of 2014 received the mean monthly values of October 2014). After the annual mean albedo was calculated, a three-year mean filter was applied to smooth the high inter-annual variability. The annual mean blue-sky albedo (BA) was calculated with the direct radiation ratio and annual mean BSA/WSA for each year. We used the MODIS Level-3 atmosphere monthly global product (MOD08M3, Collection 6; Platnick *et al*.^26^) to calculate the direct radiation ratio at the earth’s surface. The monthly average solar zenith angle, aerosol optical depth, cloud optical depth, cloud fraction and water vapor were used to calculate the direct radiation ratio using the libRadtran^27^. The following values were used as entries in the radiative transfer simulations: solar zenith angle (0°–80°, at 10° intervals), Cloud Optical Depth (1, 2, 3, 5, 10, 20, 30, 40, 50, 60, 70, 80, 90, 100), Aerosol Optical Depth at 550 nm (0.01, 0.025, 0.05, 0.1, 0.2, 0.3, 0.4, 0.5, 0.6, 0.7, 0.8, 0.9, 1.0), and water vapor (0, 15, 30, 45 mm). The direct radiation ratio was calculated at the spatial resolution of one degree and then interpolated to 500 m.

We then calculated SF for the entire study area based on all possible year combinations allowing for the calculation of SF following the equation:

1 $${\rm{SF}}=({{\rm{BA}}}_{t1}-{{\rm{BA}}}_{t2})\,\ast \,{S}_{\downarrow }$$

where BA_t1_ and BA_t2_ represent annual mean BA for years t1 and t2 (t1 < t2), respectively, and ${S}_{\downarrow }$ stands for mean downward shortwave surface flux, calculated for all years between t1 and t2. The clear-sky downward shortwave surface flux data produced by the Clouds and the Earth’s Radiant Energy System (CERES) project^28^ was used to compute ${S}_{\downarrow }$. This dataset was derived from the top-of-atmosphere (TOA) irradiance, which was acquired by the sensors on board the Terra and Aqua satellites, and calibrated using a set of independent data inputs including cloud properties identified by the MODIS products^28^. Since the spatial resolution of the surface flux data is 1°, it was resampled to 500-m to reconcile with the MODIS albedo data. The SF values were calculated for all areas that experienced stand-replacing fires between 2001 and 2012 (referred to as burned forests in this manuscript) as mapped in Chen *et al*.^22^. The extracted values were averaged according to year since fire based on which a post-fire SF trajectory was established. In order to show seasonal variation in SF changes, we also generated the monthly SF trajectories following the same methodology using corresponding monthly mean BA and downward shortwave surface flux. Monthly mean total downward shortwave surface flux within the study area between 2001 and 2015 was also calculated to show the seasonal pattern of incoming solar radiation.

### Albedo trajectories

With the available datasets, the maximum range of the SF trajectories was 14 years. However, by using the 24-year stand age map, post-fire albedo dynamics can be assessed over a longer period. Therefore the albedo trajectories within all known burns as a function of time since burning were constructed and compared. In this analysis, BSA rather than BA was used to represent albedo because of very high correlation between annual mean BSA and WSA within the study area during 2001–2015 (R^2^ = 0.99, Supplementary Fig. S1). Three BSA trajectories (i.e. mean annual, snow-off and snow-on BSA) were established for all stand-replacing fires which occurred between 1989 and 2012 using all high-quality MODIS albedo data for 2001–2015. Snow-off BSA was calculated as the mean value for June, July and August and snow-on BSA was calculated based on October and March (because during these two months there are sufficient high-quality albedo observations in the region) in each year. BSA values for September, April, and May were not used in the mean snow-on and -off calculations because the extent of snow on the ground during these months varies and thus, if included in the averaging, would represent mixed signals for both snow-on and snow-off conditions. While no MODIS-based albedo data exists for fire-induced forest loss before 2001, we used 2001–2015 albedo values for those fire events to extend the BSA trajectories of burned forests beyond the MODIS 15-year record. For example, for stand-replacing events of 1989 and 2001, BSA values of 2015 were used to assess impacts on albedo 12 and 26 years since fire, respectively. The pre-fire albedo values for stand replacing fires that occurred between 2001 and 2012 were utilized as the reference indicating the pre-fire baseline albedo dynamics. In addition to the post-fire BSA values, the pre-fire BSA values were also tracked for all burned forests and their corresponding “year since fire” values in the generated trajectories are negative.

Subsequently, the BSA anomalies for forests that suffered stand-replacing fires in each year between 2001 and 2012 were calculated and examined. BSA anomalies were calculated within the mapped burns in each of the 12 years beginning with January 1 through 2001–2015 on an 8-day basis to minimize the influence of low-quality observations due to cloud cover. Similarly, mean BSA for each 8-day cycle across 15 years (i.e. 2001–2015) was calculated for all forests that did not experience stand-replacing fires between 2001 and 2012. The BSA anomalies were then computed by subtracting each of the 8-day mean BSA for the forests that did not burned during 2001–2012 from the corresponding 8-day burned BSA for each year.

### Region-wide surface forcing assessment

Finally, we examined the spatial distribution of SF for the entire study area calculated between 2001 and 2013, which reflects the combined effect of all stand-replacing fires that occurred between 2001 and 2012. To summarize the findings, we divided the study area into 6 zones, each corresponding to 10° in longitude and 7.5° in latitude. We further compared SF for burned and unburned forests between 2001 and 2012 and all forests within each zone accounting for fraction of area burned. To aid the interpretation of revealed patterns of the spatial distribution of SF, the daily snow cover for each pixel within the study area between 2001 and 2015 was tracked using the MODIS MOD10A1 (Collection 6) daily snow cover product^29^. Once a pixel switched from snow-on to snow-free and remained so for 7 consecutive days, the first day of this 7-day window was considered the snow melt date for that pixel in that year. Due to the frequent occurrence of cloud cover, the qualified 7-day window was allowed to have no more than 5 cloudy days. The snow onset dates were calculated in a similar fashion, however, instead of searching for the snow-off windows, the algorithm searched for the snow-on windows. The first day of the identified 7-day window was considered the snow onset date for that pixel in that year. Generalized linear regression was fitted based on the snow onset and melt dates, respectively, of 150,000 (i.e. 10,000 for each year between 2001 and 2015) randomly selected sample points with respect to year.

## Results

### Fire-induced cooling effect

**Figure 1:**
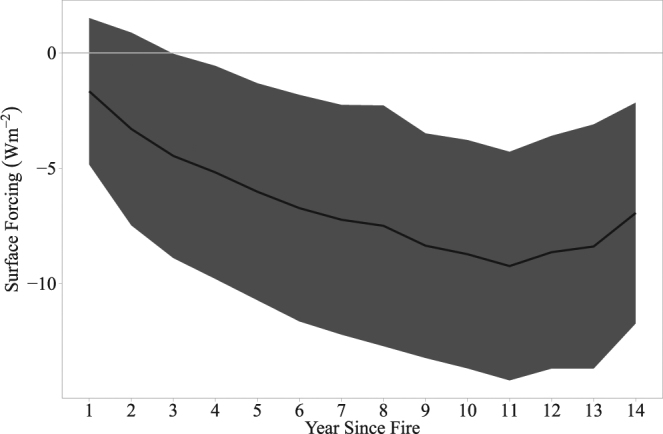
Annual mean SF trajectory for burned forests. Grey area denotes ±1 standard deviation range.

**Figure 2:**
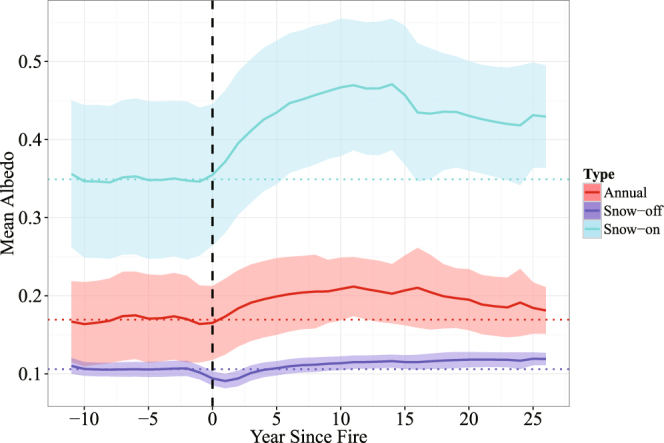
Mean BSA trajectories for the burned forests generated using the MODIS albedo data for 2001–2015. The vertical dashed line represents the year of fire occurrence. The negative “year since fire” range represents pre-fire conditions. The horizontal dashed lines represent the mean pre-fire BSA for the corresponding trajectories. Uncertainties are represented by shaded areas within ±1 standard deviation.

**Figure 3:**
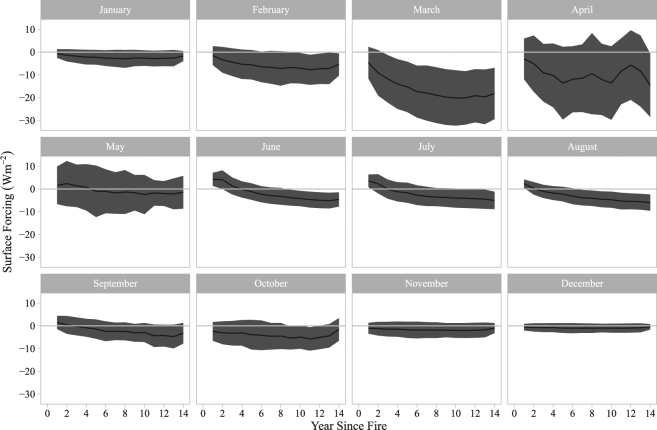
Monthly SF trajectories for the burned forests. Uncertainty is represented by ±1 standard deviation.

**Figure 4:**
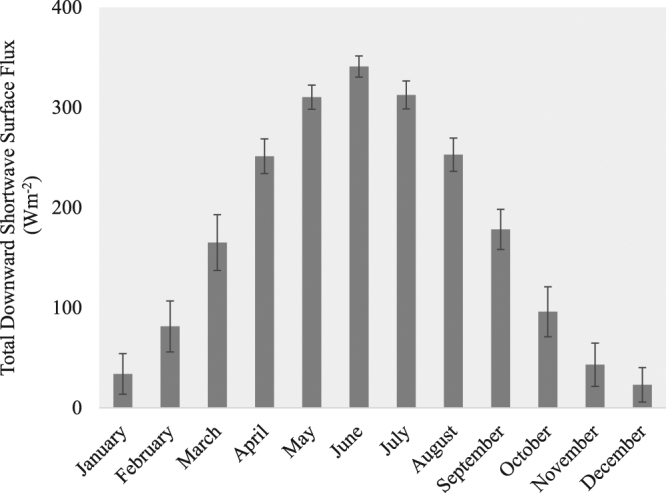
Monthly mean total downward shortwave surface flux between 2001 and 2015. Error bars represent ±1 standard deviation.

The analysis of the SF trajectory for stand-replacing fires that occurred only between 2001 and 2012 (and thus can be linked directly to post-fire MODIS albedo observation at the annual time step) establishes that the mean annual SF of the burned forests is consistently below zero after fire for at least 14 years (which is the maximum temporal range that the SF trajectory could reach with the available datasets). During the first year after the fire event, the mean SF is slightly negative (−1.69 ± 0.01 Wm^−2^). The cooling effect, however, grows rapidly and continuously until the 11^th^ year when it peaks at −9.60 ± 0.03 Wm^−2^, after which it gradually lessens to reach −7.15 ± 0.03 Wm^−2^ by year 14 (Fig. 1). The BSA trajectories (Fig. 2) show that the mean albedo of the burned forests remained elevated compared with the pre-fire level for 26 years, suggesting that the cooling effect of the burned forests persisted during that period. In terms of monthly distribution of SF (Fig. 3), March shows the strongest post-fire cooling, followed by April. The cooling exists in all other months as well, but the magnitude is much smaller. In comparison, the monthly mean downwards shortwave surface flux exhibits a bell-shaped pattern with a peak in June (Fig. 4).

### Mechanism and longevity of cooling effect

**Figure 5:**
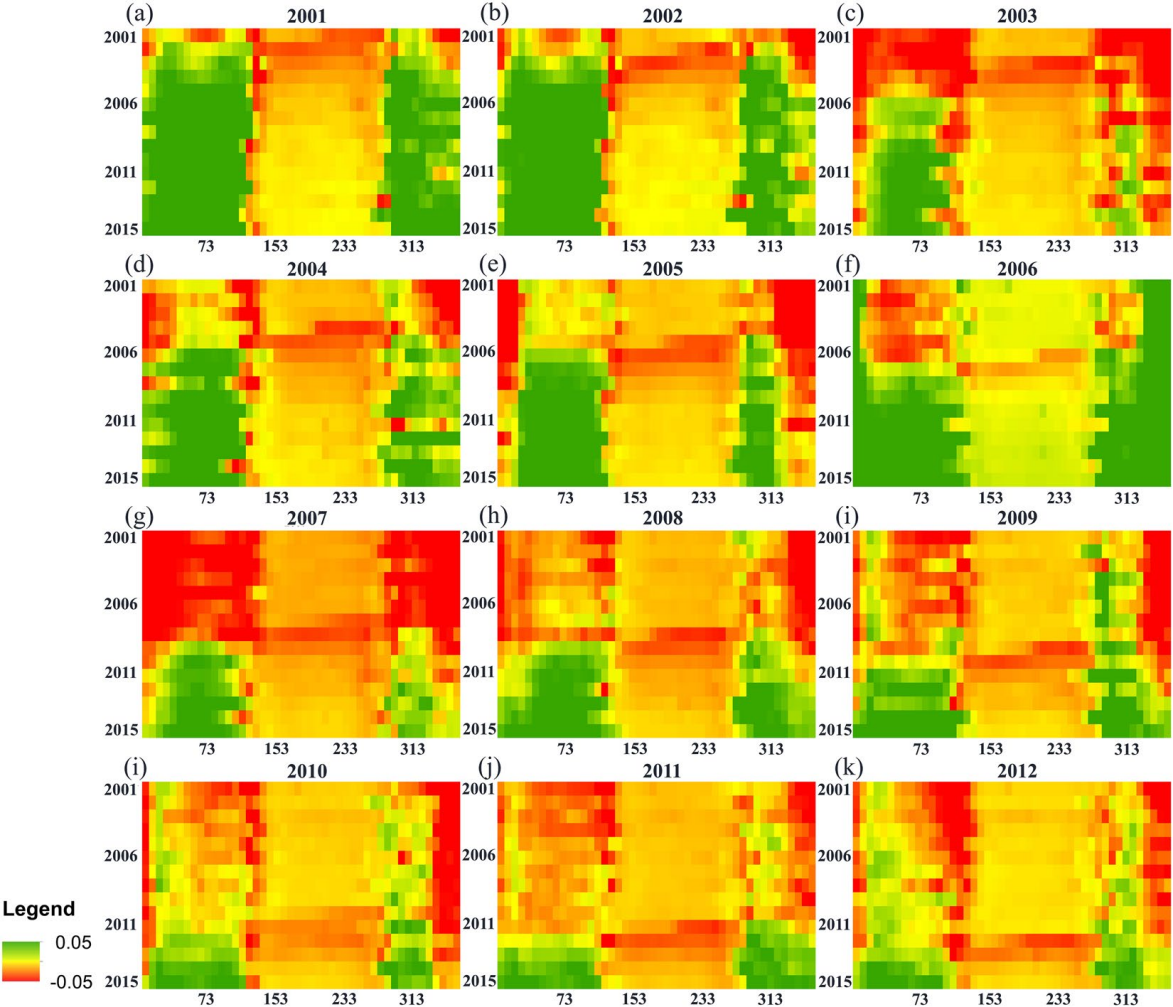
The 8-day BSA anomalies calculated for the forests that burned between 2001 and 2012 (represented by letters (**a**–**k)** in chronological order). Green and red represent albedo increase and decrease, respectively, relative to the forests that did not experience stand-replacing fires during 2001–2012. In each graph, the x-axis is Julian Day and the y-axis is year (from 2001 to 2015). The graphs show the increases in albedo mostly occur during the snow-on periods and the year after which albedo starts to increase closely follows the fire events.

Figures 2 and 5 reveal the mechanism of the observed cooling effect in the burned forests. Annual mean albedo of the burned forests (Fig. 2) increased considerably immediately after fire, and the increase was especially notable in the beginning and the end of each year (Fig. 5), which together correspond to the snow season in the region. An inter-comparison between three trajectories (mean annual, snow-off and snow-on BSA) in Fig. 2 reveals several interesting patterns. First, all three albedo trajectories show an overall increase in surface albedo after fire, but the magnitude of the increase is different, with the snow-on albedo being the most significant (up to an increase of 0.12 in year 14 relative to the mean pre-fire level). Second, the increase in mean snow-on BSA takes place immediately after fire, whereas mean snow-off BSA shows an initial decrease the first year after fire, after which albedo begins to increase. Similar to mean snow-on BSA, the annual mean BSA trajectory begins increasing immediately after fire, which is likely because snow-on BSA has a stronger control over annual mean BSA than snow-off BSA. Third, the snow-off trajectory shows a small but seemingly continuous increase up to year 26, whereas the annual mean and winter trajectories reach maximum levels in year 11, followed by observable decreases. Despite the decreases, by year 26, annual mean and snow-off BSA is still at elevated levels compared with the pre-fire conditions.

### Spatial distribution of 12-year SF

**Figure 6:**
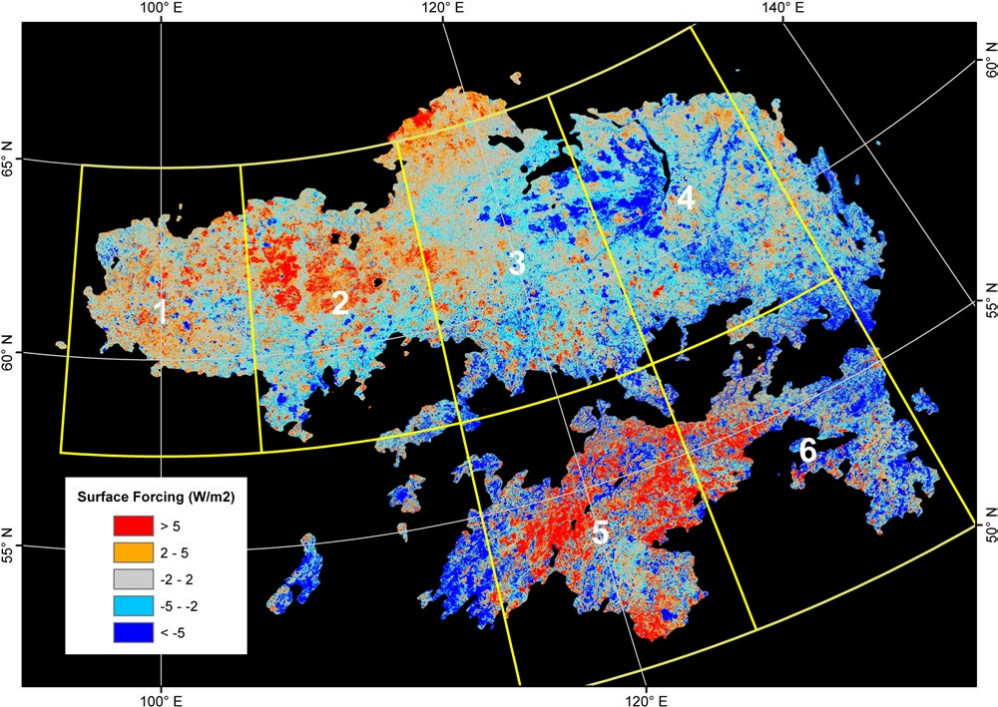
SF between 2001 and 2013 for the Siberian larch forests. Black color represents unclassified area or missing data. Six 10° × 7.5° zones are superimposed on top of the distribution map and are labeled sequentially. The map is created in ArcGIS 10.4.1^48^ (https://www.arcgis.com/).

**Figure 7:**
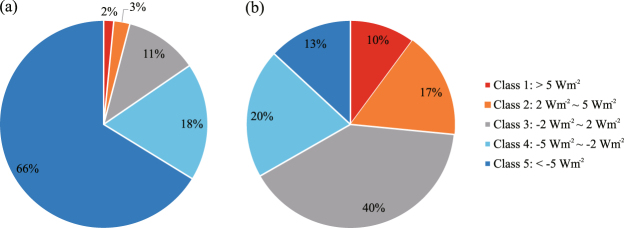
Area proportion of the five SF (Wm^−2^) classes within (**a**) burned and (**b**) unburned forests between 2001 and 2012.

**Figure 8:**
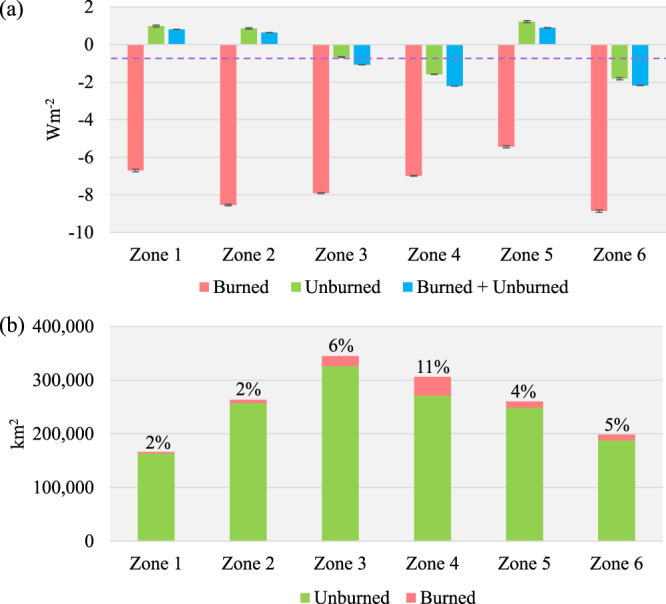
Results of the analysis which was based on 6 10° × 7.5° zones (labeled in Fig. 6): (**a**) Mean 2001–2013 SF calculated for all 6 zones based on burned forests only, unburned forests (between 2001 and 2012) only and burned and unburned forests combined. The dashed line represents mean 2001–2013 SF calculated based on all forests (burned + unburned) across the entire study area. Uncertainties are represented by ±1.96 × standard error. (**b**) Areal distribution of burned and unburned forests across the 6 zones. Labels show the proportions of burned forest area out of all forests area in the corresponding zones.

**Figure 9:**
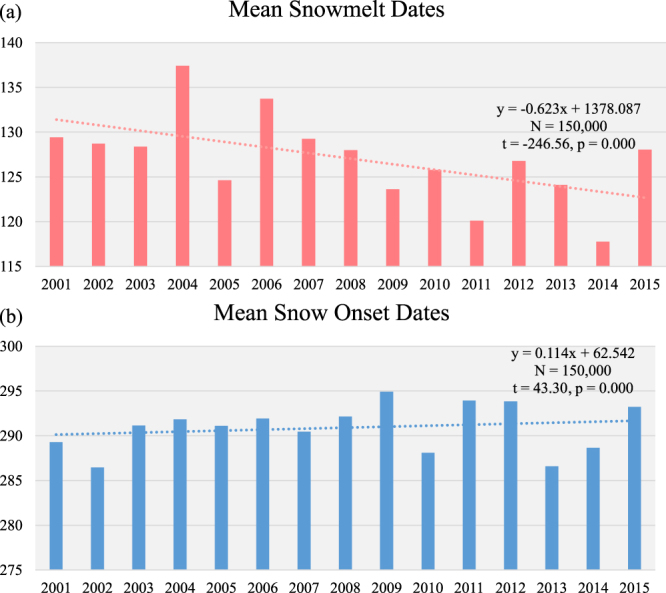
Mean snowmelt and snow onset dates in the study area between 2001 and 2015 determined based on the MODIS MOD10A1 daily snow cover product. The dashed lines represent linear trends.

The spatial distribution of albedo-induced SF calculated for 2001–2013 across the study area is heterogeneous (Fig. 6). After classifying the total range of SF into 5 classes (1: SF > 5 Wm^−2^, 2: 2 Wm^−2^ < SF < 5 Wm^−2^, 3: −2 Wm^−2^ < SF < 2 Wm^−2^, 4: −5 Wm^−2^ < SF < −2 Wm^−2^, 5: SF < −5 Wm^−2^), it appears that the distribution of areas with significant cooling effects (SF < −2 Wm^−2^) is consistent with the areas that experienced stand-replacing fires between 2001 and 2012. This is well illustrated through the comparison in Fig. 7: within the burned forests, 84% of the area consists of the two cooling classes (Classes 4 and 5), whereas the warming (Classes 1 and 2) and cooling (Classes 4 and 5) effects take up similar proportions within the unburned forests. The zonal analysis further confirms the dominance of the cooling effect of the burned forests over the region. Despite the fact that the net SF of the entire study area between 2001 and 2013 is estimated to be −0.78 Wm^−2^, the net 2001–2013 SF for three (i.e. Zones 1, 2 and 5) out of six zones is found to be positive (Fig. 8a). We believe this is a combined effect of two causes: (1) these three zones have the three lowest burned area proportions (Fig. 8b) and (2) the net SF of the forests that did not experience stand-replacing fires is positive. A closer examination on these three zones reveals that the warming effect of the unburned forests is likely caused by a decrease in albedo during the transitional period between the snow-on and snow-off seasons (i.e., Julian Days 113–145). The analysis of snow melt and onset dates (Fig. 9) shows that over the 15 years, the snow melt in the entire region occurred earlier and the trend is statistically significant (t = −246.56, p = 0.000). During the same time, the mean snow establishment was delayed (t = 43.30, p = 0.000), although the magnitude of the delay is much smaller. The changes in mean snow melt and establishment dates together lead to a general lengthening of snow-free seasons since 2001 by 10–15 days (Fig. 9).

## Discussion

In a recent study, Rogers *et al*.^14^ have shown that the albedo-induced cooling effect caused by wildfires in Northern Siberia is much smaller than that in North America, with the net climatic impact of wildfires in Siberia potentially resulting in a small warming or neutral effect. However, it is important to consider that this observed pattern reflects only present-day or even very recent past fire regimes in North America and Siberia. As mentioned in the introduction, in contrast to the dominance of stand-replacing fires within the North American forests^21^, stand-replacing fires in Siberia represent a much smaller proportion of the total estimated fire activity^7,30^. However, even within the last 30 years, early evidence of an increase in the amount of stand-replacing fire in Siberia has emerged^22^. Coupled with the expected further increase in extent and particularly severity of fire events under the projected climate change in boreal ecosystems^31^, stand-replacing fire events in Siberia are likely to play a more prominent role in influencing the Earth’s radiation budget compared to the current conditions and should be modeled explicitly moving forward. Our study aims to augment the results of Rogers *et al*.^14^ by offering an isolated evaluation of the albedo-induced forcing of stand-replacing fires within the Siberian larch forests which, in terms of fire ecology, are more compatible with those in the North American boreal forests. The SF and albedo trajectories established for the forests that burned between 2001 and 2012 showed that stand-replacing fires in this region impose a strong cooling effect for at least 26 years after fire and the magnitude is directly comparable to that reported for North America^14^. According to our results, the peak SF of stand-replacing fires on average exceeds −9 Wm^−2^.

In addition to examining stand-replacing fires separately from surface fires, this study used different input datasets for estimating both burned area and surface albedo which likely also contributed to the differences in our and Rogers *et al*.^14^ estimates. The stand age map that we used to provide the locations of the stand-replacing fires was derived based on a series of Landsat-based remotely sensed datasets^32,33^. Its spatial resolution is 30 m which may have translated into better delineation of stand-replacing fires than the MODIS-based burned area product analyzed by Rogers *et al*.^14^. In addition, the Collection 5 MCD43A3 albedo product, used in the 2015 assessment, has been reported to suffer from the degradation of MODIS Terra sensor^34^. We encountered the impact of sensor degradation in a preliminary analysis, where the cooling effect of the burned forests estimated using the Collection 5 data was up to 2 Wm^−2^ smaller compared to estimates derived from the Collection 6 MODIS albedo product, which was corrected for the inconsistency and used in the current study. Moreover, the CERES downward shortwave surface flux data that we used is more accurate than the reanalysis dataset^35^ used by Rogers *et al*. in terms of both spatial resolution (i.e. 1° vs. 2.5°) and production basis (i.e. observational vs modeled).

The similarity in the magnitude of the albedo-induced cooling effects of stand-replacing fires between the Siberian larch forests and the North American boreal forests likely reflects a common driver of cooling immediately after fire: forest canopy removal. The post-fire albedo anomalies between 2001 and 2015 (Fig. 5) and the seasonal albedo change patterns (Fig. 2) identify the elevated snow season albedo, which results from canopy removal, as the primary driver of the observed cooling effect within the burned Siberian forests. However, over a longer term the North American and Siberian SF trajectories within burns, which are determined largely by the successional forest recovery and different species composition, are likely to diverge. In most North American boreal forests, there usually is a period of time at the early successional stages when forest stands are dominated by deciduous broadleaf species^13,16,36,37^. As a result, forest albedo is typically higher than pre-fire and remains elevated for a considerable amount of time. According to Liu and Randerson^17^, it may take more than 60 years for forest albedo to recover to the pre-fire level in the black spruce forests in Alaska. In the Siberian larch forests, however, there is typically no secondary succession, and larch remains the dominant genus very early in the burned sites^38–40^. As a result, the duration of the fire-induced cooling is primarily determined by the time it takes for the larch stands to reach pre-fire levels of canopy closure. The results suggest that the forest canopy in the study area does not recover fully within at least 26 years, which is consistent with several field-based estimates of forest recovery in the Siberian larch forests^40–42^. According to Zyryanova *et al*.^40^, larch forests reach maturity around 50 to 90 years after a disturbance. However, ecological maturity is a more complex term than canopy closure, which may be reached sooner during the recovery process.

Although post-fire forests show strong cooling effects caused by the overall increase in albedo, it is worth noting that there is a small warming effect induced by the initial decreases in summer albedo represented by the purple dashed line in Fig. 2. This may be due to the char deposition on the forest floor and boles, a consequence of combustion. With time char is gradually removed and within 4 years the summer albedo exceeds the pre-fire level.

The seasonal variability in solar radiation input also influences the post-fire SF (Figs 3 and 4). While surface albedo within burned forests is the highest during the snow-on season, the total solar radiation input is very low from November through January, and subsequently, the cooling effect is small. However, as the solar input grows rapidly from February through April - the spring part of the snow-on season - the cooling effect reaches its annual peak. The magnitude of the spring cooling effect is ultimately large enough to drive the mean annual SF values to ~ −9 Wm^−2^ (Fig. 1).

The spatial patterns of albedo-induced SF between 2001 and 2013 vary across the Siberian larch forest region (Fig. 6). The comparison of area proportions of the five SF classes between the burned and unburned forests (Fig. 7) and a zonal analysis examining the 12-year SF in six different subsets of the entire study area (Fig. 8) show that areas with significant cooling effects (i.e. SF < −2 Wm^−2^) generally coincide spatially with stand-replacing fires. Within burned forests, the cooling effect dominates (Fig. 7a), whereas in forests that did not experience stand-replacing burns between 2001 and 2012, warming and cooling effects are mixed in roughly similar proportions (Fig. 7b). Proportionally, the unburned forest area outweighs the stand-replacing burns across the entire region (Fig. 8b) and thus, overall, the total SF of each zone is only slightly lower than the SF of unburned forests.

There are multiple potential explanations for the considerable existence of cooling in the unburned forest group. First, within the forests that did not burn between 2001 and 2012, there is a considerable proportion that experienced stand-replacing fires during the past several decades before 2000, including those that have been estimated to be burned in the pre-2000 component of the stand age map^22,43^. And since it has been shown that forest albedo is higher than the pre-fire level over at least 26 years since fire, the inclusion of these older burned forests results in the appearance of significant cooling effect. Second, the regional pattern also includes impacts from surface fires which were not examined in this study. It is generally accepted that surface fires occur extensively in the Siberian larch forests^7,21,30,38,44,45^, although there is a large uncertainty regarding both their spatial extent^46^ and climatic impacts. However, we hypothesize that they could also impose a cooling effect because damages to the understory could also result in an increase, albeit smaller, in snow-on albedo. Finally, the uncertainty of the stand age map could also have caused the latter forest group to show negative forcing.

The spatial analysis of the net 12-year SF in the Siberian larch forests reveals a general warming effect which particularly noticeable within the three zones (1, 2, and 5) which were least impacted by stand-replacing fires between 2001 and 2012 (Fig. 8). We attribute the overall warming to the observed decrease in albedo during the transitional periods between the snow-on and snow-off seasons, which is, in turn, a consequence of a considerable lengthening of snow-free period (Fig. 9). Between 2001 and 2015, snow melt in this region occurred earlier in the season at an average rate of −0.6 days year^−1^, while snow establishment occurred later at an average rate of 0.1 days year^−1^. These transitional periods are particularly important as the amount of incoming solar radiation is large and, thus, the total impact of surface albedo on SF is very high. This implies that continuous warming predicted for region will further augment the positive forcing imposed by undisturbed boreal forests as shown by Randerson *et al*.^11^ and offset the cooling effect induced by wildfires. However, since fire activity is also expected to increase under the projected warming^47^, the net albedo-induced forcing of the whole Siberian larch forests would hinge on the interaction between the fire-induced cooling effect and the climate-induced warming effect. Further studies aimed at improving our understanding of the wildfire occurrence, the long-term post-fire trajectories of surface albedo and SF, and other ecological (e.g. shifts in species composition) and environmental (e.g. the length of snow-free season) changes within the Siberian larch forests are needed to quantify the overall impact of wildfire on regional and global climate system.

## Electronic supplementary material

Supplementary Figure
