# Supplementary material for: Strong cooling induced by stand-replacing fires through albedo in Siberian larch forests
Source: Sci Rep. 2018 Mar 19;8:4821. doi: 10.1038/s41598-018-23253-1 (PMC5859174; doi:10.1038/s41598-018-23253-1)
Supplement: Supplementary file 1 — Supplementary Figure [file 41598_2018_23253_MOESM1_ESM.pdf]

Supplementary Figure for

Strong cooling induced by stand-replacing fires through albedo in Siberian larch forests

Dong Chen[1*], Tatiana V. Loboda[1], Tao He[2], Yi Zhang[1], Shunlin Liang[1]

1. Department of Geographical Sciences, University of Maryland, College Park, Maryland, USA

2. School of Remote Sensing and Information Engineering, Wuhan University, Wuhan, Hubei, China

* Corresponding author (Tel: +1-7062019830, Email: itscd@umd.edu)

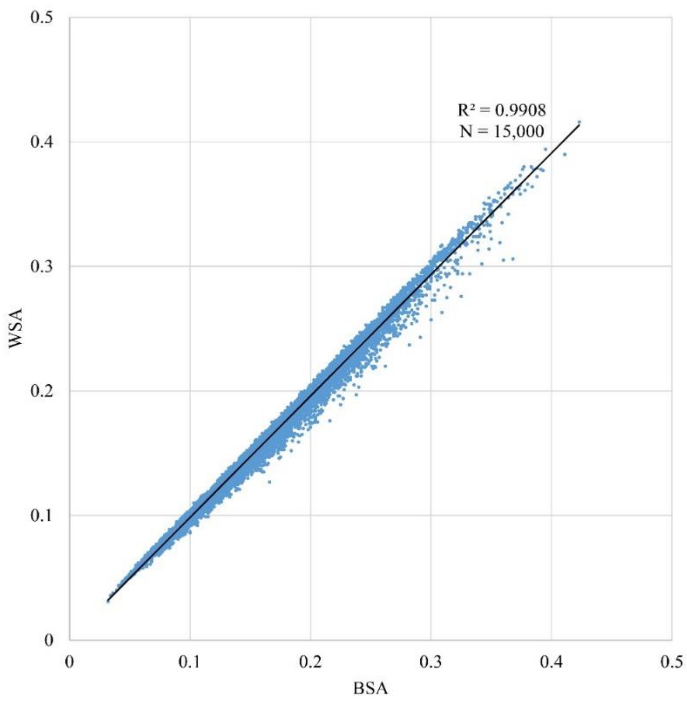

Supplementary Figure S1. The statistical relationship between annual mean BSA and WSA between 2001 and 2015 within the study area.
